# Pathophysiological impact of CXC and CX3CL1 chemokines in preeclampsia and gestational diabetes mellitus

**DOI:** 10.3389/fcell.2023.1272536

**Published:** 2023-10-19

**Authors:** Amin Ullah, Jing Zhao, Rajeev K. Singla, Bairong Shen

**Affiliations:** ^1^ Joint Laboratory of Artificial Intelligence for Critical Care Medicine, Department of Critical Care Medicine, Institutes for Systems Genetics, Frontiers Science Center for Disease-Related Molecular Network, West China Hospital, Sichuan University, Chengdu, China; ^2^ School of Pharmaceutical Sciences, Lovely Professional University, Phagwara, Punjab, India

**Keywords:** preeclampsia, gestational diabetes mellitus, CXC chemokines, fractalkine (CX3CL1), inflammation, therapeutic discoveries

## Abstract

Diabetes-related pathophysiological alterations and various female reproductive difficulties were common in pregnant women with gestational diabetes mellitus (GDM), who had 21.1 million live births. Preeclampsia (PE), which increases maternal and fetal morbidity and mortality, affects approximately 3%–5% of pregnancies worldwide. Nevertheless, it is unclear what triggers PE and GDM to develop. Therefore, the development of novel moderator therapy approaches is a crucial advancement. Chemokines regulate physiological defenses and maternal-fetal interaction during healthy and disturbed pregnancies. Chemokines regulate immunity, stem cell trafficking, anti-angiogenesis, and cell attraction. CXC chemokines are usually inflammatory and contribute to numerous reproductive disorders. Fractalkine (CX3CL1) may be membrane-bound or soluble. CX3CL1 aids cell survival during homeostasis and inflammation. Evidence reveals that CXC and CX3CL1 chemokines and their receptors have been the focus of therapeutic discoveries for clinical intervention due to their considerable participation in numerous biological processes. This review aims to give an overview of the functions of CXC and CX3CL1 chemokines and their receptors in the pathophysiology of PE and GDM. Finally, we examined stimulus specificity for CXC and CX3CL1 chemokine expression and synthesis in PE and GDM and preclinical and clinical trials of CXC-based PE and GDM therapies.

## 1 Introduction

After 20 weeks of gestation, preeclampsia (PE), a severe pregnancy complication, is primarily characterized by hypertension and proteinuria. The global incidence of PE ranges between 3% and 10% (Steegers et al., 2010). PE, which occurs approximately in 3%–5% of pregnancies worldwide, is a significant contributor to maternal and fetal morbidity and mortality, high healthcare expenses, and up to 25% of low-birthweight preterm infants (Mol et al., 2016; Robillard et al., 2019). Hence, PE endangers the safety of both mother and infant during the perinatal period. Nonetheless, the etiology and pathogenesis of PE remain unclear, and the typical recommended treatment strategy is still inadequate. The previous description of the onset and progression of PE’s clinical symptoms can be described as follows (Huppertz, 2008; Duley, 2009): Stage 1 is when the mother and the allogeneic embryo do not have complete immune tolerance for each other when an embryo is implanted (Chaiworapongsa et al., 2014); Stage 2 of PE is described by placental hypoxia and syncytiotrophoblasts (STBs) dysplasia, which establishes the pathological basis for PE by resulting from diminished trophoblast invasion in the first trimester; and stage 3 is defined by placental ischemia and hypoxia, which result in severe metabolic problems, improper spiral artery remodeling, placental apoptosis, and an imbalance in the immune response (Redman et al., 2014). Thus, a maternal immune system’s instability is a critical component in the development of PE.

Gestational diabetes mellitus (GDM) affects women between 24 and 28 weeks of pregnancy and can adversely affect their health. According to estimates, pregnant women (aged 20–49) with some form of diabetes delivered 21.1 million live babies in 2021 (Guariguata et al., 2022). Globally, the incidence of GDM has rapidly increased in recent years, creating a severe issue for public health (Szmuilowicz et al., 2019).

Most studies found a correlation between GDM and PE in singleton pregnancies, indicating that better GDM management and medication may reduce the occurrence of PE, which is very advantageous to improving pregnancy outcomes. As a consequence, enhancing GDM management and therapy may lower PE occurrence. Table 1 shows the correlated studies among GDM and PE.

**TABLE 1:** Correlation studies among GDM and PE.

Study type/Disease	Investigated markers	Study content	Results/Outcome	References
*In vivo*/GDM/PE	KISS1 and KISS1R	Association	Increased KISS1 and KISS1R expression in GDM may contribute to the altered placentation process and the emergence of PE	Kapustin et al. (2019)
*In vivo*/GDM	Serum vitamin D	Risk	Significantly increased risk of GDM development in pregnant women with low vitamin D level, which increases the chance of PE onset	Al-ajlan et al. (2018)
*In vivo*/GDM/PE	DNA methylation patterns	Association	Human placenta DNA methylation patterns are consistently and strongly correlated with PE and GDM	Liu et al. (2014)
*In vivo*/GDM/PE	BMI and blood glucose levels	Association	In singleton pregnancy, GDM is independently correlated with PE	Yang and Wu (2022)
*In vivo*/GDM/PE	DEGs	Association	These findings suggest that STB patients with GDM may have severe early-onset PE due to inadequate endometrial infiltration, angiogenic dysfunction, and oxidative stress	Sun et al. (2018)
*In vivo*/GDM/PE	Blood glucose and other related markers during pregnancy	Association	GDM and PE were positively correlated in the analysis	Mistry et al. (2021)
*In vivo*/GDM/PE	Endothelial dysfunction/blood biospecimens	Association	GDM closely associated with the development of PE	Mcelwain et al. (2020), Ladd-acosta et al. (2023)
*In vivo*/GDM/PE	IL-6, TNF-α, and leptin	Risk	GDM mothers cause fetal overgrowth. Obese females with hypertension, like PE, have reduced placental vascular branching	Howell and Powell (2017)
*In vivo*/GDM	IGF in placenta tissue	Association	GDM is linked to PE, type I and II diabetes	Rout and Lulu (2019)
*In vivo*/GDM/PE	BMI and glycemic profile	Association	GDM is also linked to innate immune response upregulation, which may cause vascular dysfunction and health issues, one of PE’s pathophysiologic causes	Sunjaya and Sunjaya (2018)
*In vivo*/GDM	Urine albumin-to creatinine ratio	Association	Elevated UACR in GDM suggested PE in this investigation	Wong et al. (2014)

Previous research has demonstrated the critical roles that maternal immune regulators, such as the chemokines CXC and CX3CL1 (fractalkine), play in the interaction between PE and their surroundings (Liu et al., 2015; Lei et al., 2019; Szewczyk et al., 2021). Furthermore, Liu et al. (2022) indicated the connection between GDM and CXC chemokines. It is essential to consider these chemokines as potential targets or biological markers in the pathogenic process that precedes the development of PE and GDM (Liu et al., 2015, 2022). According to studies, PE and GDM have high levels of CXC chemokine expression, and there is a direct link between their expression and the pathophysiology of PE and GDM. Therefore, improving PE and GDM patients’ survival rates requires understanding the relationship between these parameters (CXC and CX3CL1), PE, and GDM progression. The roles of the chemokines CXC and CX3CL1 in PE and GDM have been intensively studied; however, the pathogenesis of PE and GDM’s underlying mechanisms is still unknown. Therefore, in this review, we will discuss how CXC and CX3CL1 chemokines affect PE and GDM and how they might be used to treat them.

Chemokines are transcriptional, low-molecular-weight peptides (8–10 kDa) primarily chemotactic for leukocytes (Baird et al., 2011). Based on the number of amino acids between the first and second cysteine residues in the peptide sequence, Chemokines are divided into four subfamilies (C, CC, CXC, and CXXXC) where X is any residue of an amino acid (Zlotnik and Yoshie, 2000). The chemokine family consists of two key subgroups, CXC and CC subtypes (Kawaguchi et al., 2019). CXC chemokines are short secretory proteins containing four highly conserved cysteine amino acid residues and one non-conserved amino acid residue separating the first two cysteines (Taub and Oppenheim, 1994). Depending on whether or not the NH2-terminus contains the Glu-Leu-Arg (ELR) motif, representatives of the CXC chemokine family are classified into two categories: ELR+/ELR-members (Dhawan and Richmond, 2002). The ELR + includes CXCL1 to CXCL3 and CXCL5 to CXCL8 members. The ELR-members are CXCL4, CXCL9 to CXCL14, and CXCL16 (Strieter et al., 1995; Lee et al., 2018; Li et al., 2020b).

In 1997, Bazan et al. (1997), Pan et al. (1997) published the first descriptions of the chemokine CX3CL1. On human chromosome 16, CX3CL1 includes three additional amino-acid residues in addition to the first two cysteine residues. The sole member of the CX3C (delta) subfamily is CX3CL1 (Stievano et al., 2004). Non-hematopoietic chemokine CX3CL1 has two different forms: a transmembrane protein with a long, mucin-like stalk attached to the chemokine domain and a soluble peptide liberated from the cell surface through proteolytic cleavage (Bowen et al., 2002). Human CX3CL1’s soluble chemokine domain attracts monocytes, T cells, and natural killer (NK) cells but not neutrophils. CX3CL1 membrane-bound functions include promoting leukocyte linkage and adhesion and activating target cells. Many inflammatory diseases, including asthma, rheumatoid arthritis, and osteoarthritis, have elevated levels of soluble CX3CL1, which is responsible for leukocyte, NK cell, and T-cell chemotaxis in inflammation. Therefore, in contrast to chemotaxis, the primary function of CX3CL1 is immune system modulation (D’Haese et al., 2012). CX3CL1, the sole endogenous ligand that CX3CR1 binds to, exhibits considerable therapy potential (Lu et al., 2022a). Table 2 displays the leading role of CXC chemokines in the progression and development of PE and GDM.

**TABLE 2:** CXC chemokines with their main role during PE and GDM.

CXC chemokine/CX3CL1	Disease	Expression	Main role	References
CXCL1 and CXCL8	PE	↑	leukocyte activation and inflammation	Mellembakken et al. (2001a), Faulhaber et al. (2011), Han et al. (2015)
CXCL2 and CXCL5	PE	↑	inflammation	Giambrone et al. (2019)
CXCL3	PE	↓	shallow implantation or abnormal placentation	Gui et al. (2014), Liao et al. (2022)
CXCL5	PE	↑	macrophages activation	He et al. (2023)
CXCL5, CXCL6, and CXCL8	PE	↑	vascular infiltration of neutrophils in preeclamptic women	Walsh et al. (2022)
CXCL8	PE	↑	placental inflammation	Valencia-Ortega et al. (2019), Ozmen et al. (2022)
CXCL9 and CXCL10	PE	↑	anti-angiogenesis, intravascular inflammation and fetal growth restriction	Gotsch et al. (2007), Moreno-eutimio et al. (2014), Darakhshan et al. (2019b), Li et al. (2020a), Kiyokoba et al. (2022)
CXCL11	PE	↑	Th-1 cells recruitment inflammation	Boij et al. (2012), Miralles et al. (2019), Accortt et al. (2023)
CXCL12/CXCR4	PE	↑	migration of dMSCs	Lei et al. (2019)
CXCL12/CXCR4/CXCR7	PE	↓	trophoblastic cells apoptosis	Lu et al. (2016)
CXCR12/CXCR4	PE	↑	inhibit apoptosis of HTR-8/SVneo cells	Yang et al. (2023)
CXCL13	PE	↑	inflammation	Shin et al. (2010)
CXCR5	PE	↑	massive chemotaxis, Tfh cells and autoantibodies	Heydarlou et al. (2018), Fu et al. (2023)
CXCR5	PE	↑	systemic inflammation	Heydarlou et al. (2018)
CXCL14	PE and GDM	↑	potentially regulate trophoblast outgrowth	Lekva et al. (2016)
CXCL16	PE	↑	liver damage and inflammation	Tok et al. (2019), Li et al. (2021)
CXCL16	PE and GDM	↑	dyslipidemia and systemic inflammation	Lekva et al. (2017)
CXCL16	PE	↑	vascular inflammation	Estensen et al. (2015)
CXCR2	PE	↑	Inflammation	Wang et al. (2019b)
CXCR2	PE	↓	impairing trophoblast invasion	Wu et al. (2016)
CX3CL1	PE	↑	inflammation	Siwetz et al. (2015), Huang et al. (2019), Lekva et al. (2020)
CX3CL1	PE	↑	underdevelopment of placental vascular network	Szewczyk et al. (2021)
CXCL1, CXCL3, and CXCL12	GDM	↓	reduced angiogenic potential	Chen et al. (2020)
CXCL1, CXCL8-10, and CXCL12	GDM	↑	lung dysfunction and inflammation	Li et al. (2020c), Zhang et al. (2022b), Liu et al. (2022), Pascoe et al. (2022)
CXCL3, CXCL5, CXCL6 and CXCL14	GDM and PE	↑	islets inflammation	Agarwal et al. (2019), Halvatsiotis et al. (2019)
CXCL8	GDM	↑	omental adipose tissue inflammation	Dong et al. (2022)
CXCL10	GDM	↑	placental inflammation	Pan et al. (2021a)
CXCL9 and CXCL10	GDM	↑	regulate inflammatory pathways	Wang et al. (2019a)
CXCL15	GDM	↑	inflammation	Qin et al. (2022)

The ↑ arrow shows upregulation, and the ↓ arrow shows downregulation.

**FIGURE 1:**
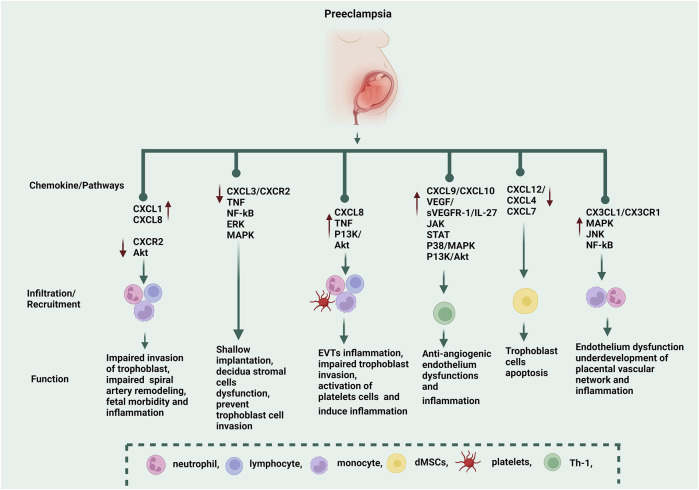
The functions of CXC and CX3CL1 chemokines in the progression of PE. CXCL1 and CXCL8 recruit neutrophils, monocytes, and lymphocytes, while CXCR2 is downregulated during PE via the AKT pathway, and these CXC chemokines induce impaired invasion of the trophoblast, impaired spiral artery remodeling, fetal morbidity, and inflammation. Through the TNF, NF-kB, ERK, and MAPK pathways, CXCL3/CXCR2 causes shallow implantation, decidua stromal cell dysfunction, and trophoblast cell invasion. CXCL8 recruits neutrophils, lymphocytes, monocytes, and platelets via TNF and P13K/Akt pathways, which induce extravillous trophoblasts (EVTs) inflammation, impaired trophoblast invasion, and activation of platelet cells (which induce inflammation). CXCL9 and CXCL10 are anti-angiogenic chemokines. CXCL10 recruits Th-1 via the IL-27/JAK, P38/MAPK, P13K, and Akt pathways, which induce inflammation. CXC10 is correlated to vascular growth factor (VEGF/sVEGFR-1), and the excessive secretion of these molecules induces endothelium dysfunction. CXCL12, CXCR4, and CXCR7 recruit decidual mesenchymal stem cells (dMSCs), which induce trophoblast cell apoptosis. CX3CL1/CX3CR1 recruits monocytes and neutrophils via MAPK, JNK, and NF-kB pathways, which induce endothelium dysfunction and inflammation. CX3CL1 was negatively correlated to the vascular/extravascular tissue index, which indicated that CX3CL1 was involved in the underdevelopment of the placental vascular network. ↑ The red arrow symbol shows upregulation, while the ↓ arrow shows downregulation.

PE and GDM have inflammation as a defining feature. Numerous studies have shown that inflammation is crucial to the pathogenesis of PE and GDM (Yin et al., 2014; Nguyen-ngo et al., 2020a). Most CXC chemokines are inflammatory (Liong et al., 2018). Fakhr and others (Fakhr et al., 2022) reported that placental villi exhibit a preeclamptic-like phenotype due to the inflammatory mediator TNF-α. Furthermore, a disruption in the nuclear factor kappa B (NF-kB) signaling pathways balance in patients with PE results in aberrant placental trophoblast cell death (Aban et al., 2004; Caballero et al., 2013). A study found that CXCL3/CXCR2 participates in the tumor necrosis factor (TNF) and NF-κB signaling pathway significantly and is downregulated in PE (Rong et al., 2021), which indicated that the abnormal expression of CXCL3 induces decidual stromal cells dysfunction. In addition, Huang and others (Huang et al., 2019) reported that in the presence of excessive proinflammatory stimuli such as TNF-α, interleukin-1 beta (IL-1β), and interferon-gamma (IFN-γ), signaling through mitogen-activated protein kinase 1/2 (MAPK1/2), jun N-terminal kinase (JNK), and NF-kB pathways induces CX3CL1/CXCL10 production (Figure 1). Thus, it is likely that early pregnancy in women results in higher tissue levels of these chemokines, eventually developing PE. In addition, PE vascular smooth muscle cells treated with interleukin-17A (IL-17A) produced more neutrophil-related chemokines, such as CXCL8, CXCL5, and CXCL6. These chemokines cause inflammation (Walsh et al., 2022), which indicates that IL-17A inflammatory stimuli could be responsible for the vascular infiltration of neutrophils in PE. Furthermore, *in vitro*, Nguyen-ngo et al. (2019), Nguyen-ngo et al. (2022) used human placental and adipose tissue samples. They treated them with TNF and lipopolysaccharides (LPS) to induce a GDM-like environment. TNF and LPS treatment significantly increased the expression of inflammatory CXC chemokines such as CXCL1/5/8/9/10, which indicated that TNF and LPS were inflammatory stimuli that stimulated the proinflammatory chemokines in GDM. Thus, inflammatory stimuli like IL-1-β, TNF-α, IL-1, IFN-γ, and LPS can induce the expression and synthesis of CXC and CX3CL1 chemokines even though these inflammatory chemokines are not constitutively expressed (Pinheiro et al., 2013; Huang et al., 2019; Nguyen-ngo et al., 2022; Walsh et al., 2022). However, as shown in Table 3, a single stimulus may only impact a small number of particular chemokines.

**TABLE 3:** Specificity of the stimulus for CXC and CX3CL1 chemokines expression and synthesis during PE and GDM.

Chemokines	Stimulus	References
CXCL3/CXCR2	TNF	Rong et al. (2021)
CXCL2	IL-1β	Huang et al. (2006)
CXCL3	IFN-γ	Rong et al. (2021)
CXCL5	IL-17	Walsh et al. (2022)
CXCL6	IL-17	Walsh et al. (2022)
CXCL8	IL-17, IFN-γ, IL-1β, and IL-27	O’Haraa et al. (2010), Li et al. (2011), Pinheiro et al. (2013), Rong et al. (2021), Walsh et al. (2022)
CXCL9	IFN-γ	Gotsch et al. (2007)
CXCL10	IFN-γ and IL-27	Gotsch et al. (2007), Yin et al. (2014)
CXCL11	IFN-γ	Gotsch et al. (2007)
CX3CL1	TNF-α, IL-1β, and IFN-γ	Huang et al. (2019)
CXCL1/5/8/9/10/12	TNF and LPS	Nguyen-ngo et al. (2019), Nguyen-ngo et al. (2022)

Successful pregnancy and health depend on the homeostatic balance between systemic and local pro- and anti-angiogenic factors (Rana et al., 2012). In addition, numerous studies have demonstrated that the pathophysiology of PE depends on the appropriate balance between angiogenic and antiangiogenic mediators (Molvarec et al., 2010). Vascular endothelial growth factor (VEGF), which has six isoforms—VEGF-A, B, C, D, and placental growth factor (PlGF)—is the primary regulator of angiogenesis (Heinke et al., 2012). These mediators bind to and activate VEGFR-1, 2, and 3 (Karaman et al., 2018). The binding patterns of VEGF-A and VEGFR-2, VEGF-B, and PlGF are specific to VEGFR-1, while VEGF-C and VEGF-D bind to VEGFR-3 and impact the lymphangiogenic pathway (Karaman et al., 2018).

Zhang et al. (2019) reported that increased tumor growth factor-beta (TGF-β) levels from decidual Treg cells inhibited dNK cell function by decreasing IFN-γ/CXCL8/CD107a expressions. They found considerable positive relationships between IFN-γ and CD107a expression in dNK during preterm and term pregnancy but not in PE. PE had a strong negative connection between VEGF and CD107a expression, showing that dNK’s angiogenic and cytotoxic capabilities are conditionally regulated during pregnancy. Additionally, there is a correlation between GDM-PE and VEGF expression in clinical studies. Compared to the standard group, the GDM-PE group has different levels of VEGF and receptors. Moreover, the PE and GDM-PE groups had higher levels of fms-like tyrosine kinase-1 (FLT-1) expression (Dong, 2019; Krishnasamy et al., 2019; Sugimoto et al., 2019; Ren et al., 2020; Al-Ofi et al., 2021; Alqudah et al., 2021; Atallah et al., 2021; Bolatai et al., 2022). These results indicated that the upregulation of VEGF/FLT-1 in GDM-PE induces endothelium dysfunction. Thus, we consider VEGF and its receptors linked to these two pregnancy problems. The two diseases may be correlated because endothelial damage impacts VEGF and its receptor in GDM and PE. Together, both diseases have comparable cumulative risks. VEGF and its receptor alterations cause aberrant blood vessel proliferation and persistent hypoxia.

It is believed that VEGF imbalance precedes maternal-fetal complement progression. PE is caused by maternal pathogenesis preceding the placenta (Ying and Hongyan, 2020; Liu et al., 2021). VEGF antagonistic receptor irregularities are typically the primary symptom of a predisposition to PE. This receptor is often called soluble FLT-1 (Cindrova-Davies et al., 2011). It is one of the direct causes of the symptomatic proteinuria and hypertension that accompany PE in pregnancy. In general, an increase in sFLT-1 will combine with other endothelial factors to hinder binding, diminish normal placental villi infiltration and the capacity to stimulate vascular growth, and worsen vascular activity throughout the PE process during pregnancy. As a result, alterations in VEGF and its associated tyrosine kinase receptors can be observed in pregnancy problems (Bolatai et al., 2022). However, more case data is needed to demonstrate the link between VEGF and its receptors and GDM and PE mentioned in earlier studies.

Several studies have linked PE to higher levels of inflammatory signals and lower levels of immune regulatory markers. A Mexican study found significantly lower levels of regulatory T cells (Tregs) in the serum of women with varying degrees of PE severity. Increased levels of inflammatory signals such as interleukin-6 (IL-6), TNF-α, and interleukin-8 (IL-8), as well as anti-angiogenesis chemokines such as CXCL10 and CXCL9, were found among those with severe PE (Moreno-eutimio et al., 2014). In addition, Gotsch et al. (2007) also noted that CXCL10 exhibits anti-angiogenic behaviors, which are hallmarks of PE. They suggest that increased maternal serum concentrations of CXCL10 could contribute to establishing an anti-angiogenic state when combined with sVEGFR-1 because the anti-angiogenic effects of the serum from pregnant women with PE can be reversed in the endothelial cell tube formation bioassay whenever VEGF and PIGF are administered (Maynard et al., 2003). The administration of sVEGFR-1 to pregnant animals can also result in clinical symptoms of PE, such as proteinuria and hypertension (Maynard et al., 2003). Therefore, the authors speculate that increased maternal CXCL10 concentrations, in conjunction with sVEGF-R1, may aid in the establishment of an anti-angiogenic state in the context of their finding that CXCL10 exhibits anti-angiogenic properties, which is a unique feature of PE (Figure 1) (Gotsch et al., 2007).

Dijmărescu et al. (2021) describe the angiogenic and anti-angiogenic factors as helpful indicators for PE diagnosis and prognosis. Compared to the mild PE group, VEGF and CXCL10 concentrations in the severe PE group showed a stronger correlation with indices. The serum levels of CXCL10 were statistically higher in the severe PE group than in the mild PE group, demonstrating a substantial association between VEGF and CXCL10. The results showed that VEGF and CXCL10 were the biomarkers that best corresponded with indices that assessed the severity of PE.

CX3CL1 can promote integrin-dependent trophoblast migration and induce angiogenesis through the VEGF/hypoxia-inducible factor-1 alpha (HIF-1α) pathway (Hannan et al., 2006; Ryu et al., 2008; Szukiewicz et al., 2014). There is evidence that angiotensin II deregulation of CX3CL1 occurs in early-onset PE. As a result, it may play a role in the proinflammatory trophoblast-monocyte interaction (Siwetz et al., 2015; Nonn et al., 2019). Szewczyk et al. (2021) discovered considerably higher CX3CL1 serum levels and placental expression in PE patients and a negative correlation with the vascular/extravascular tissue index. The authors postulated that severe underdevelopment of the placental vascular network in PE is associated with alterations in CX3CL1 expression and distribution.

## 2 Role of CXC chemokines in the pathogenesis of PE

### 2.1 CXCL1/CXCR2

The secretion of CXC chemokines, such as CXCL1 and CXCL8, was dramatically increased when placental trophoblast cells were exposed to high doses of glucose (25 and 50 mM) (Han et al., 2015). These findings suggested that first-trimester trophoblast cells were in a pro-inflammatory state because of the excessive glucose deposition. These data show the trophoblast’s pleiotropic response to hyperglycemia, which may help to explain the strong association between diabetes and PE.

Due to reduced trophoblast invasion and aberrant vascular remodeling, it may prolong placental oxidative stress, which in turn may exacerbate the dysfunction of trophoblasts that is a defining feature of PE (Anton et al., 2013; Fu et al., 2013). The research also demonstrated that IL-1β induces CXCL1 expression, which aids trophoblast invasion (Baston-buest et al., 2017). In addition, microRNAs (miRNAs) are small, non-coding, single-stranded RNA that regulate gene expression at the post-transcriptional and transcriptional levels to a lesser extent (O’Brien et al., 2018). Hayder et al. (2021) recently revealed that overexpression of miR-210-3p lowered the mRNA levels of CXCL1 and CXCL8. These chemokines may invade extravillous trophoblasts (EVT) and enlist immune cells at the sites where the spiral arteries are remodeling during PE. These results imply that overexpression of miR-210-3p could result in impaired maternal spiral artery remodeling, which would contribute to the pathogenesis of PE, or that miR-210-3p regulates spiral artery remodeling events by inhibiting the expression of these chemokines (CXCL1 and CXCL8).

Neonatal patients, especially preterm newborns, have poor chemotaxis, which may be a factor in their increased risk of sepsis. Two chemokines are essential for leukocyte movement: CXCL1 and CXCL8 (Greer et al., 1989; Sullivan et al., 2002). Following neutrophil, monocyte, and leukocyte activation, plasma levels of CXCL1 and CXCL8 rise, which operate as immune system modulating factors (Mellembakken et al., 2001a, 2001b; Faulhaber et al., 2011). During PE, the numerous activations of chemokines (CXCL1 and CXCL8), which turn on neutrophils, monocytes, and leukocytes in the fetus, may affect fetal morbidity. In addition, the CXCL1 methylated gene is implicated in the pathogenesis of PE (Jia et al., 2012).

Wu et al. (2016) observed that CXCR2 mRNA and protein expression levels were considerably lower in PE placentas than in normal controls. The authors also showed that low levels of CXCR2 in the placenta may contribute to the pathogenesis of PE via downregulation of matrix metalloproteinase-2 (MMP-2) and matrix metalloproteinase-9 (MMP-9) via the Akt pathway, which impairs the invasion of the trophoblast (Wu et al., 2016). These results suggest that lower levels of CXCR2, which inhibit trophoblast invasion by decreasing MMP-2 and 9, may play a role in the development of PE. Furthermore, recent studies demonstrated significant abnormalities/downregulated in CXCR2 expression during PE (Wang et al., 2019b; Meeuwsen et al., 2020; Zhang et al., 2020). Further research elucidating the pathways that involve CXCR2 signaling, such as the Akt pathway, may provide new knowledge on the impact of CXCR2 in PE patients.

### 2.2 CXCL2

In a recent study, Ma et al. (2023) extracted decidual stromal cells from PE patient tissue and transfected them with miR-455-3p inhibitors to study their function. The study discovered that miR-455-3p inhibitor transfection raised CXC chemokines such as CXCL2, CXCL3, and CXCL8. The translation level was also examined by assessing CXC chemokine expression in decidual stromal cell-derived culture media (CM). Transfection of miR-455–3p inhibitors increased CXCL2, CXCL3, and CXCL8 expression in decidual stromal cells-derived CM. These findings demonstrate that the downregulation of miR-455-3p enhances CXCL2, CXCL3, and CXCL8 production in decidual stromal cells. In addition, Zhou et al. (2023) revealed that in PE decidual stromal cells, downregulation of miR-92a enhances the production of CXCL2, CXCL3, and CXCL8. Interferon regulatory factor-3 (IRF3) may control the levels of CXC chemokines like CXCL2, CXCL3, and CXCL8 in the decidual stromal cells of PE directly or indirectly. miR-92 may bind to the 3′UTR of IRF3, promote its downregulation, and eventually regulate the secretion of CXC chemokines in these cells. These CXC chemokines are overexpressed in PE patients, which causes inflammation. Thus, the latest studies indicated that miRNAs play a vital role in the pathophysiology of PE in decidual stromal cells.

CXCL2, responsible for monocyte and macrophage chemoattraction and activation, was also elevated in PE placental tissue. According to a study, IL-1 dramatically increases CXCL2 mRNA and protein expression in first-trimester decidual cells (Huang et al., 2006). Likewise, references indicated that CXCL2 is upregulated in PE patients and the blood pressure high/5 (BPH/5) female mouse model of PE (Yan et al., 2013; Batts et al., 2021). Interestingly, Johnston and others (Johnston et al., 2021) observed that CXCL2 mRNA expression was considerably lower in PE hepatic transaminases low platelets (HELLP) mouse model syndrome. The decreasing trend of CXCL2 during late pregnancy is unknown. To determine if these inflammatory mediators’ expression changes during normal gestation, comparing these mouse strains to other mouse strains or whether the BPH/5 female mouse represents a model of fatty liver disease associated with pregnancy rather than HELLP syndrome will be necessary.

### 2.3 CXCL3/CXCR2

Another crucial proinflammatory cytokine, CXCL3, attracts neutrophils and macrophages, which aids in developing inflammation-related colorectal adenoma (Payne and Cornelius, 2002). CXCL3 attracts and activates CXCR1 and CXCR2 receptors, which are present on the surface of endothelial, epithelial, and colloid cells. Consequently, this is crucial for the natural development of inflammation and angiogenesis (Russo et al., 2014). Coincidentally, these modules are also very closely related to the emergence of PE. More importantly, a previous study found that exogenous CXCL3 protein may boost trophoblast migration and proliferation, and placental CXCL3 levels in the severe PE group were significantly lower than in the mild PE and regular groups, suggesting that CXCL3 is pathologically linked to severe PE (Wang et al., 2018). Plasma CXCL3 levels and placental CXCL3 expression significantly differed in women with severe PE. CXCL3 had a role in trophoblast invasion, which showed that it was involved in shallow implantation (Gui et al., 2014). The placental expression levels of CXCL3, which are detected in STBs and vascular endothelium, were lowered in cases of severe PE. *In vitro*, HTR-8/SV-Neo cell invasion and proliferation were both induced by exogenous CXCL3. In addition, a recent bioinformatics study revealed that CXCL3/CXCR2 are involved in the TNF signaling and the NF-kB signaling pathway and that CXCL3 is strongly expressed in normal decidual macrophages but dramatically downregulated in PE patients (Rong et al., 2021), as a result, the aberrant expression of these differentially expressed genes (DEGs) (CXCL3) has an impact on the dysfunction of the decidual stromal cells, which revealed the interactions between immune cells in the decidua.

Additionally, Liao et al. (2022) discovered that demethylation (TARID), a new long non-coding RNA (lncRNA) linked to PE, plays a part in the onset and progression of PE by modulating the CXCL3, ERK, or MAPK pathways. TARID stimulates trophoblast movement, invasion, and tube formation. The downregulation of TARID expression during PE mediates the CXCL3, extracellular signal-regulated kinase (ERK), or MAPK pathways, which may prevent trophoblast invasion and spiral artery remodeling by impairing cell growth, invasion, and tube formation (Liao et al., 2022).

### 2.4 CXCL6

A recent bioinformatic study found that the hub gene (most closely associated with disease) CXCL6 functions as a PE biomarker and prediction model in patients (Lin et al., 2022). MicroRNA (MiR-101) was shown to be considerably reduced in PE placentas and was found to be involved in the regulation of several apoptotic proteins under endoplasmic reticulum stress (Zou et al., 2014). MiR-101 also suppresses trophoblast HTR-8/SVneo cell migration and invasion by targeting CXCL6 in PE (Zhang et al., 2021).

The Y153H, C allele of the transcription factor STOX1 (STORKHEAD-BOX1 PROTEIN 1) has been associated with the early utero-vascular etiology of placental diseases as well as the onset or progression of PE (Pinarbasi et al., 2020; Dunk et al., 2021). A subsequent study by the same group raises the possibility that the STOX1 Tyr153His mutation in the extravillous trophoblast may contribute to preterm birth and PE. Utero-vascular remodeling is a compromised process that preterm birth and PE share (Dunk et al., 2021). The researchers used primary EVT explant, placental decidual coculture models, and EVT-like cell lines to transfect the variant for functional validation. EVT and EVT-like cells transfected with the STOX1 Tyr153His mutation released lower IL-6 and IL-8 but higher levels of CXCL6 and tumor necrosis factor-related apoptosis-inducing ligand (TRAIL) than control wild-type EVT cells and EVT-like cells. Some of these genes, like CXCL6, were higher in the endothelial cells of mice that overexpressed STOX1A. They were linked to inflammation and cellular stress (Miralles et al., 2019), indicating STOX1A’s functional significance in the development of PE.

### 2.5 CXCL8

In a bioinformatic study, the Kyoto Encyclopedia of Genes and Genomes (KEGG) pathway enrichment analysis revealed that CXCL8 was upregulated via phosphatidylinositol 3-kinase (PI3K)/protein kinase B (AKT) signaling pathways in PE, which might assist inflammation and impaired trophoblast invasion during PE (Jiang et al., 2020). In addition, interferon-stimulated gene 15 (ISG15) serves as a crucial regulator of proinflammatory cytokine expression in EVTs during PE, and HTR8/SVneo cells with silencing of ISG15 had elevated levels of CXCL8 mRNA (Ozmen et al., 2022).

TNF-α stimulates the release of CXCL8 and other proinflammatory cytokines by endothelial cells (O’Haraa et al., 2010). There is evidence that TNF-α levels in maternal serum from PE-complicated pregnancies are higher than in normotensive pregnancies (Szarka et al., 2010). Shaw and others (Shaw et al., 2016) found that TNF-α was a significant factor in endothelial cells releasing and activating the proinflammatory cytokine CXCL8. This suggests that blocking TNF activity could help reduce the effects of maternal endothelial dysfunction in PE.

During the development and progression of PE, platelets may promote blood coagulation and initiate inflammation. Platelets may also play a role in the inflammatory response in PE (Wang et al., 2017). However, in preeclamptic women, the degree of inflammation is related to the degree of platelet activation. This tends to be linked to processes that alter cytokine levels, including CXCL8 (Sahin et al., 2014).

Pinheiro and his team discovered that women with severe PE had more CXCL8 in their blood than pregnant women with normal blood pressure. In the same study, they discovered a significant correlation between CXCL8 and IFN-γ in severely preeclamptic individuals and postulated that this could play a role in the pathophysiology of PE (Pinheiro et al., 2013). Alteration in chemokine levels, such as CXCL8, may, in our opinion, directly impact the endothelium of the maternal systemic vasculature. Furthermore, studies found higher CXCL8 serum concentrations in PE patients, implying that CXCL8 plays a role in the pathogenesis of PE (Gigraci et al., 2010; Garcia et al., 2018; Valencia-Ortega et al., 2019). Hence, targeting CXCL8 through developing PE therapeutic techniques may result in significant clinical applications in PE. CXCL8 may function as a biomarker for PE patients.

### 2.6 CXCL9, CXCL10, and CXCL11

CXCL9, CXCL10, and CXCL11 mRNA expression in the chorioamniotic membranes from PE was also higher in chronic chorioamnionitis cases than in those without chronic chorioamnionitis (Kim et al., 2010). Another comparative study reported that CXCL9 and CXCL10 were associated with inflammation in PE patients, which were found to be increased in PE patients (Brien et al., 2020).

IFN-γ-induced Th1 cytokine milieu also releases these CXC chemokines (Gotsch et al., 2007). CXCL10 and CXCL11 appear to be part of a Th1-like inflammatory response in PE, with CXCL10 potentially more reliable as a biomarker in PE patients (Boij et al., 2012).

According to Yin et al. (2014), IL-27 may have a critical role in PE. IL-27 was reported to increase CXCL10 expression in trophoblastic cells via activating the Janus kinase/signal transducers and activators of transcription (JAK/STAT), p38 MAPK, and PI3K-Akt signaling pathways (Figure 1). Understanding and treating PE might improve through elucidating the connections between IL-27 and CXCL10. Prior research has shown that women with PE have higher concentrations of CXCL10 and CXCL9 (Oliveira et al., 2011; Haedersdal et al., 2013; Chedraui et al., 2015; Kalinderis and Papanikolaou, 2015; Darakhshan et al., 2019b; Li et al., 2020a; Dijmărescu et al., 2021).

### 2.7 CXCL12 and CXCL16

Regarding the function of CXC12, CXCR4, and CXCR7 in PE, reports are inconsistent. Prior studies have documented elevated levels of CXCL12 in the placenta of patients with PE compared to the average control population (Schanz et al., 2011; Hwang et al., 2012). Nevertheless, several studies have demonstrated that the placentas of patients with severe PE have downregulated levels of CXCL12 and its receptors, CXCR4 and CXCR7 (Kim et al., 2014; Lu et al., 2016). CXCL12 has also been shown in further investigations to reduce the apoptosis rate of term trophoblast cells (Lu et al., 2016, 2020). Therefore, the downregulation of CXC12, CXCR4, and CXCR7 may interfere with trophoblast apoptosis and contribute to the development of severe PE. Interestingly, pregnant women with PE had greater levels of CXCL12 in their mid-trimester amniotic fluid; however, the precise mechanism is unknown (Tseng et al., 2009). Moreover, CXCL12/CXCR4 signaling influences several disorders linked to pregnancy, especially PE (Ao et al., 2020).

It has been demonstrated that bone morphogenetic protein 9 (BMP9) regulates the processes involved in endothelial dysfunction (Long et al., 2015). Yang and others (Yang et al., 2023) revealed that serum from PE patients had lower levels of CXCL12 and CXCR4 expression and that there was a positive correlation between BMP9 and these expression levels. In HTR-8/SVneo cells, overexpression of BMP9 increased the levels of CXCL12 and CXCR4. By activating the CXCL12/CXCR4 pathway, BMP9 promoted the migration and invasion of HTR-8/SVneo cells and inhibited apoptosis. It seems that BMP9 might be a biomarker for PE because it controls the CXCL12/CXCR4 axis. Lei et al. (2019) found that the CXCL12/CXCR4 axis can mediate the migration behavior of decidua-derived mesenchymal stem cells (dMSCs). dMSCs can migrate to the decidua layer along a CXCL12 concentration gradient in patients with PE, suggesting that it may play a role in the development and progression of PE. Finally, these data imply that the CXCL12/CXCR7/CXCR4 axis may be a critical molecular insight into PE that needs further investigation. Remarkably, in PE, the chemokines also exhibit their effects on decidual immune cells (Valencia-Ortega et al., 2019).

In STOX1 mice with PE, Miralles et al. (2019) examined the cytokine plasma profile and discovered aberrant CXCL16 expression. However, findings showed that CXCL16/CXCR6 expression was similar in PE patients and average pregnant women (tested at 35 weeks) (Schanz et al., 2011). One possible explanation for these conflicting results is that the tests were conducted at various gestational weeks. In addition, CXCL16 levels in the blood increased considerably in PE women at weeks 22–24, 30-32, and 36–38. In contrast, peripheral blood mononuclear cells (PBMC) mRNA expression of CXCL16 decreased (Lekva et al., 2017). Furthermore, numerous studies showed that the CXCL16 levels are significantly greater in PE patients (Estensen et al., 2015; Panagodage et al., 2016; Tok et al., 2019; Shi et al., 2020; Li et al., 2021). To date, no study has directly reported that aberrant CXCL16 chemokine expression is the underlying etiology of PE. These findings, taken together, demonstrate aberrant CXCL16 expression in the PBMC of PE patients; however, CXCL16 expression at the maternal-fetal interface during PE has not yet been investigated. Therefore, the fundamental mechanisms remain unknown. CXCL16 is overexpressed at the maternal-fetal interface and has biological processes, such as promoting trophoblast invasion (Zhang et al., 2022a). PE can be induced by inadequate trophoblast invasion and uterine spiral artery remodeling (Wang et al., 2020a). Hence, it is feasible that abnormal CXCL16 expression is connected to PE. However, further reliability and validity research are needed to reveal the specific mechanism.

## 3 Role of CX3CL1 chemokine in the pathogenesis of PE

Previous studies showing the role of CX3CL1 in the feto-maternal interaction in the process of placental invasion during PE indicated that CX3CL1 may be associated with missed abortion (Hannan et al., 2014; Usta et al., 2018)**.** In a recent study, significantly increased content of CX3CR1+ cells in the decidua of PE patients and an increase of CX3CR1-positive cells may related to colonization of the decidua by monocytes (CX3CR1 was used as a marker of the macrophages of monocytic origin) attracted by inflammatory signals from circulation (Vishnyakova et al., 2021). These results reflect profound PE-associated alterations in the decidua, including the pronounced endothelial dysfunction attraction of the CX3CR1-positive effector cells.

According to Kudryashova et al. (2019), there is a correlation between low levels of CD62L+, CD99^+^, CXCR1+ neutrophils, serum CXCL8, and adversely affected blood vessel elastic properties. In addition, another study found that CX3CR1+ monocyte and neutrophil levels were higher in PE patients (Panova et al., 2018). These findings clarified that, possibly, in PE, activated endothelial cells themselves regulate how tightly neutrophils adhere to the endothelium. One of the main mechanisms of inflammation is the interaction between the chemokine receptors expressed by the cells and the chemokines produced in the affected area.

The above studies show that CX3CL1 acts as an inflammatory mediator in various cell types involved in PE. It remains to be seen whether CX3CL1 is required for human inflammatory cell survival. Finally, several lines of evidence point out that CX3CL1 is another player in the pathogenesis of PE that could be a valuable therapeutic target. A better understanding of the mechanism and timing of CX3CL1 actions may assist with determining the kind of PE pathology that should be targeted therapeutically. The chemokines CXC and CX3CL1 regulate the onset and progression of PE through several particular pathways, shown in Figure 1.

## 4 Role of CXCLs in the pathogenesis of GDM

### 4.1 CXCL1 and GDM

In a human study, serum levels of angiogenic (CXCL1 and CXCL12) were higher in gestational diabetes mellitus mothers (GDMM). Interestingly, newborns delivered by GDMM had higher levels of CXCL1 and CXCL12 but lower levels of angiostasis (CXCL9 and CXCL10) (Darakhshan et al., 2019a). This study speculated that increased angiogenesis chemokines (CXCL1 and CXCL12) might be involved in endothelium damage in GDM patients. In addition, a recent study revealed GDM exposure in male and female offspring mice. Whereas female offspring-specific elevated levels of proinflammatory and neutrophil chemoattractants chemokine and cytokine (CXCL1 and IL-1β) in lung lavage (Pascoe et al., 2022). Due to elevated neutrophil chemoattractants (CXCL1, IL-β), this allergen exposure may result in a rise in neutrophil recruitment to the lung. These proinflammatory changes could make female offspring of GDM mothers more susceptible to lung damage and inflammation triggered by neutrophils after allergen inhalation, which could result in asthma. Furthermore, a bioinformatic study found that T1D, T2D, and GDM patients have DEGs CXC chemokines (CXCL1, 2, 3, 5, 10, and 12) and receptor CXCR4 (Evangelista et al., 2014). This suggests that the prediction of GDM, prior to its onset, should be based on multiple markers (CXC chemokines) rather than a single marker.

### 4.2 CXCL8 and GDM

Human chorionic membrane-derived stem cells (CMSCs) derived from GDM patients exhibit high levels of inflammatory indicators such as CXCL8. According to Chen et al. (2019). CXCL8 was crucial to the inflammatory process during GDM. In addition, Li et al. (2020c) recently reported that GDM women exhibited an increased risk of neonatal infection because of an abnormal placenta. Further, the high-glucose environment activated the toll-like receptor 4 (TLR4)/myeloid differentiation primary response 88 (MyD88)/NF-kB pathway in GDM placentas. It induced the secretion of the CXCL8/IL-8. Inflammation caused by GDM breaks the homeostasis in the placenta, and autophagy balance is also destroyed by maintaining cell self-renewal and homeostasis. Autophagy was increased in GDM compared with ordinary women (Li et al., 2020c). Furthermore, Zhang and others (Zhang et al., 2017) also reported that inflammatory biomarkers, including NF-kB and CXCL8, had significant differences between GDM and healthy pregnancies. This phenomenon showed that the placentas of GDM women were in an inflammatory environment. The increased inflammatory mediators might promote intraplacental inflammatory cascades by specific gene transcription or translation.

GDM increases cancer risk in children. Bioinformatic analysis showed that cancer-related pathways, such as those in cancer and acute myeloid leukemia, were significantly enriched in umbilical cord blood mononuclear cells from GDM patients. CXCL8 was the top DEG between GDM and controls. The authors found only monocyte and granulocyte DEGs enriched in the cancer pathway, with CXCL8 being the common DEG between GDM and control groups. In GDM, the number of CXCL8+ IL1B + monocytes and CD16^+^ granulocytes increased. In the high-glucose stimulation experiment, CXCL8+IL1B + monocytes also increased significantly. These findings suggest that maternal GDM reprograms fetal monocytes and granulocytes with high CXCL8 expression, which could boost teenage cancer risk (Yin et al., 2022).

### 4.3 CXCL9, CXCL10, and GDM

The bioinformatic study revealed that CXCL9/10 was significantly enriched in the TLR signaling pathway, leading to speculation that it is a crucial gene that participates in the pathogenesis of GDM by regulating the progress of the TLR signaling pathway and may thus play a critical role in the pathogenesis of GDM by regulating the inflammatory pathway (Wang et al., 2019a). In addition, studies reported that CXCL10 is upregulation associated with the birth of a macrosomic child. Together, these results suggest that CXCL10 could be used as a predictive marker for pregnancy complications in GDM (Pan et al., 2021a; Tagoma et al., 2022).

### 4.4 CXCL16 and GDM

Lekva et al. (2017) showed that in the early stages of pregnancy, the levels of CXCL16 mRNA in PBMC were lower than in healthy pregnant women. On the other hand, the levels of CXCL16 in the blood of GDM patients were higher. Furthermore, apolipoprotein B (apoB) and low-density lipoprotein cholesterol (LDL-C) positively correlated with increased levels of CXCL16. CXCL16 also works as a scavenger receptor on macrophages, boosting LDL-C oxidation and internalization (Barlic et al., 2009), which may play an important role in the inflammatory response due to lipid accumulation. Therefore, the elevation in circulatory levels of CXCL16 in patients with GDM may be associated with abnormal lipid metabolism/dyslipidemia, although the specific mechanism remains unclear. Further studies are required to explain how the expression of CXCL16 mRNA in the PBMCs of patients with GDM decreases at a certain point during pregnancy.

## 5 Role of CX3CL1 in the pathogenesis of GDM

According to array gene expression analysis, CX3CL1/CX3CR1 was consistently markedly elevated in human endothelial cells in hyperglycemia status (Schiano et al., 2019). In GDM, higher levels of CX3CL1/CX3CR1 in the placenta are linked to increased insulin resistance (IR) and an inflammatory response. This is because circulating CX3CL1 in GDM can correlate to IR markers and proinflammatory progranulin during pregnancy (Ebert et al., 2014; Schiano et al., 2019).

TLRs recognize maintained molecular patterns and play a critical role in regulating innate immune responses and inflammation by activating inflammatory mediators such as the inflammatory transcription factor NF-kB (Medzhitov, 2001). There are increased levels of TLR2 and TLR4 in PBMC from women with GDM (Kuzmicki et al., 2013). TLR activation may play a role in the proinflammatory consequences of high glucose since it significantly increases the expression of TLR2 and TLR4 in human monocytes, which in turn activates NF-kB and secretes more proinflammatory chemokines, such as CX3CL1 (Kuzmicki et al., 2013).

**FIGURE 2:**
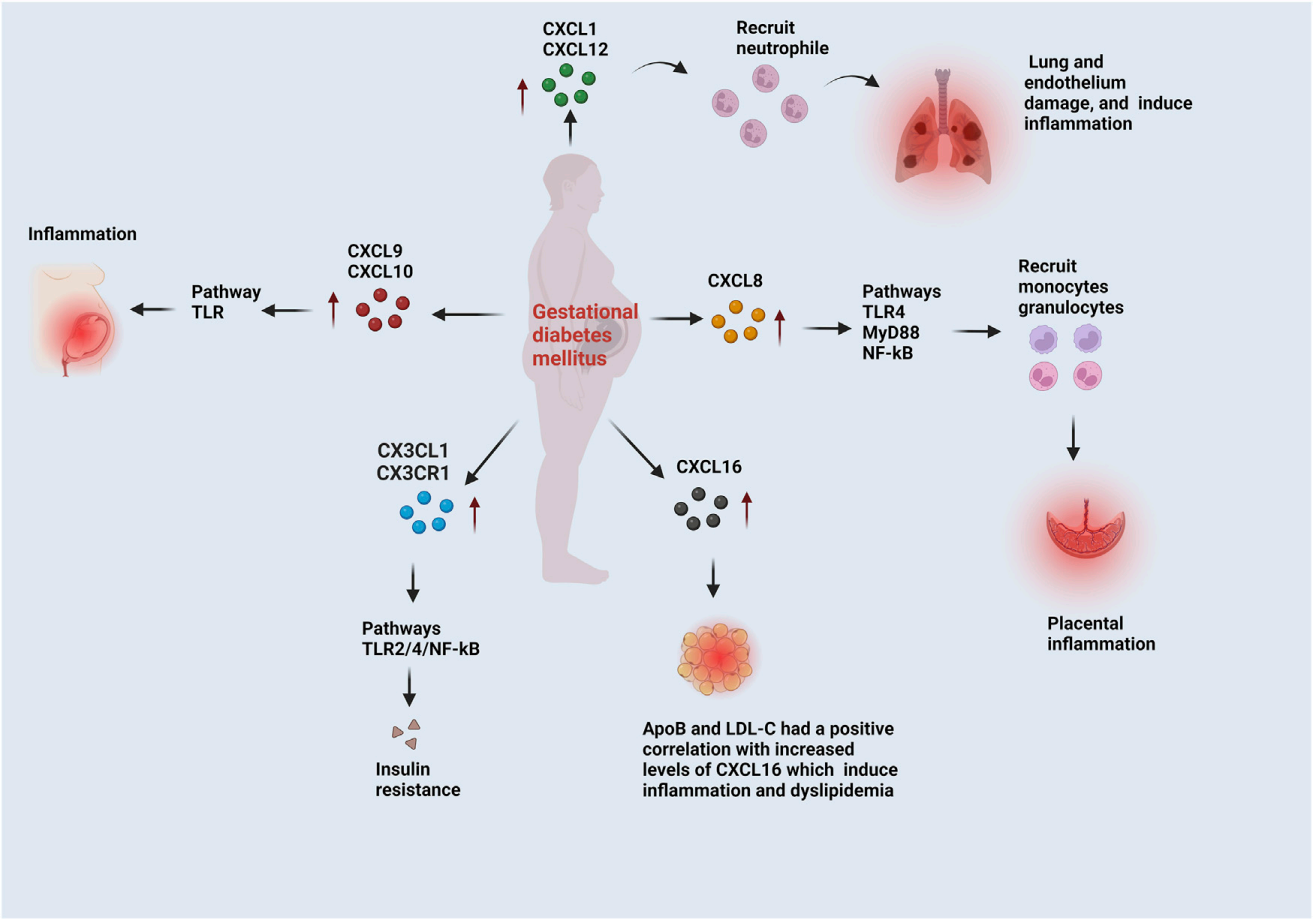
The functions of CXC and CX3CL1 chemokines in the progression of GDM. CXCL1 and CXCL12 recruit neutrophils, which induce lung and endothelium damage and inflammation. CXCL8 follows the TLR4/MyD88/NF-kB pathways and recruit’s monocytes and granulocytes, which induce placental inflammation. CXCL9 and CXCL10 induce inflammation via the TRL pathway. Adipose tissue biomarkers, like low-density lipoprotein cholesterol (LDL-C) and apolipoprotein B (ApoB), were correlated to CXCL6. These biomarkers are involved in dyslipidemia, a problem with adipose tissue. The CX3CL1/CX3CR1 is correlated to the insulin resistance markers. In GDM, CX3CL1/CX3CR1 leads to insulin resistance through the TLR2/4 and NF-kB pathways.

In the above studies, GDM patients had altered CXC and CX3CL1 chemokines. This review study summarized the most recent studies on the CXC and CX3CL1 chemokine molecular mechanisms that cause GDM pathophysiology. This enabled insights into future difficulties and possibilities for distinct clinical and therapeutic properties and consequences for GDM. The crucial role of CXC and CX3CL1 chemokines via various pathways involved in the onset and progression of GDM is illustrated in Figure 2.

## 6 Other hypertension-related disorders

### 6.1 Cardiovascular disease (CVD)

There is a documented clinically significant correlation between elevated blood pressure and cardiovascular disease (CVD) risk, including atherosclerosis and cardiac fibrosis (Perumareddi, 2019; Fuchs and Whelton, 2020). Lu et al. (2022b) reported that CXC chemokines play a vital role in the progression and development of atherosclerosis and cardiofibrosis. However, some studies reported that some CXC chemokines work as anti-CVD.

Research has indicated a negative relationship between coronary artery disease (CAD) severity and CXCL5 plasma levels. Furthermore, several aggregates of CXCL5 and CXCR2 were found in coronary atherosclerotic plaques, indicating a potential protective function of CXCL5 against CAD (Ravi et al., 2017). In addition, animal studies have demonstrated that Apoe−/− mice with CXCL5 inhibition accumulate foam cells and macrophages in atherosclerotic plaques and decrease collagen content. This finding suggests that CXCL5 could reduce the progression of atherosclerosis by preventing macrophage accumulation and foam cell formation (Rousselle et al., 2013).

Considering the substantial evidence suggesting CXCL12 had a preventive function in atherosclerosis when Apoe−/− mice were given intravenous CXCL12. Akhtar et al. (2013) noticed that although the lesion size did not alter considerably, the fibrous cap of the diseased plaques in the mice thickened, and smooth muscle cells increased. In addition, plasma CXCL12 levels were lower in advanced atherosclerotic mice than in normal mice, CAD patients, and healthy individuals. This implies that CXCL12 might have actions that prevent atherosclerosis (Damås et al., 2002; Xu et al., 2011). Furthermore, CXCL16 works as an oxLDL clearance receptor that fights atherosclerosis, and phagocytosis of oxLDL by macrophages was reduced in CXCL16-deficient animals. CXCL16^−/−^/LDLR^−/−^ mice developed accelerated atherosclerotic lesions. This evidence supports CXCL16’s antiatherosclerotic properties (Aslanian and Charo, 2006).

CXCL8 is pro-inflammatory in cardiac fibrosis, and pursuing MI-elevated CXCL9 expression stimulates fibroblast migration and proliferation. In contrast to CXCL9, CXCL10 can prevent fibroblast migration (Dobaczewski and Frangogiannis, 2009; Turner et al., 2011; Lin et al., 2019). The anti-fibrotic properties of CXCL10 and its receptor, CXCR3, prevent fibroblast migration. It may additionally play a role in lymphocyte recruitment (Frangogiannis et al., 2001; Bujak et al., 2009; Saxena et al., 2014; Li and Frangogiannis, 2021). A study found that CXCR4 antagonists could prevent cardiofibrosis in mice with type I and type II diabetes. Furthermore, CXCL12 therapy causes the proliferation and hypertrophy of rodents’ cardiac fibroblasts (CFs) and promotes CF collagen production. This outcome was more significant in hypertension models (Jackson et al., 2017; Wang et al., 2020b). Moreover, CXCL12 may promote repair and delay cardiac fibrosis. Inhibiting the scavenger receptor, CXCR7 caused the mice’s fibrosis process to slow down significantly (Chu et al., 2015). CXCL12 may promote cardiac fibrosis by attracting inflammatory cells that express CXCR4, which, in response, induces local inflammation and CF activation (Menhaji-Klotz et al., 2018).

CX3CL1 is rapidly generated following myocardial damage and chemoattracts monocytes and macrophages that express the CX3CR1 receptor (Boag et al., 2015). Since macrophages play an essential role in tissue fibrosis, it has been postulated that CX3CL1 plays a significant role in fibrotic remodeling. *In vivo* research on the CX3CL1/CX3CR1 axis’s function in fibrosis revealed that CX3CL1/CX3CR1 signaling prevents macrophage-driven fibrogenesis in a hepatic fibrosis model (Karlmark et al., 2010). While CX3CR1+ macrophages are abundant in the remodeling and infarcted myocardium (Nahrendorf et al., 2007), the significance of CX3CL1/CX3CL1 in cardiac fibrosis has not been systematically investigated. Furthermore, in a model of unilateral nephrectomy followed by angiotensin II infusion, CX3CR1 deletion did not affect myocardial fibrosis (Ahadzadeh et al., 2018).

### 6.2 Diabetes mellitus

Our recent comprehensive review study demonstrated that most of the CXC chemokines family members are upregulated during diabetes and play a vital role in the development and progression of diabetes (Ullah et al., 2023). Furthermore, numerous cell types, including fibroblasts, endothelial cells, and monocytes, release CXCL10. Patients with type 2 diabetes mellitus (T2DM) have been found to have higher levels of CXCL10 in meta-analysis research (Pan et al., 2021b). This is feasible because CXCL10 interacts with the chemokine receptor CXCR3 and TLR4 (Schulthess et al., 2009; Sajadi et al., 2013). Furthermore, CXCL10 has been linked to other processes, including stimulating T-lymphocytes and monocytes/macrophages. Additionally, clinical research has demonstrated that T2DM patients release greater levels of CXCL10 than the control group (Sajadi et al., 2013). Therefore, the interferon gamma-inducible CXCL10 is consequently believed to be a significant factor in initiating the death of b cells. CXCL10 can also reduce the viability of B cells and hinder insulin secretion. The particular process may involve the ability of the b cells stimulated by CXCL10 to maintain the activation of Akt, JNK, and cleavage of p21-activated protein kinase 2 (PAK-2), thereby altering the Akt signals from proliferative to apoptotic. It was TLR4 that mediated these effects (Tang et al., 2000; Schulthess et al., 2009).

Compared to T2DM patients, clinical investigations have demonstrated a substantial decrease in plasma CX3CL1 levels in the control group (Baldane et al., 2018). T2DM is strongly linked to CX3CL1, which is known to mediate leukocyte chemotaxis, adhesion, and survival, resulting in chronic adipose inflammation. CX3CL1 may be crucial in attracting monocytes to adipose tissue, which subsequently causes IR and systemic inflammation (Cefalu, 2011). The mechanism of leukocyte chemotaxis and adhesion mediated by CX3CL1 may impact T2DM and adipocyte dysfunction.

Finally, the studies described above suggested that hypertension-related disorders such as PE, GDM, CVDs, and T2DM alter specific CXC chemokines, including CXCL5, CXC10/CXCR3, CXCL12/CXCR4, CXCL16/CXCR6, and CX3CL1/CX3CR1. These CXC chemokines play a critical role in anti-CVDs, whereas in PE, GDM, and T2DM, they cause inflammation, the anti-angiogenesis process, damaged trophoblastic cells, dyslipidemia, and IR. Additional research is necessary to clarify the mechanisms underlying the interactions between CXC chemokines and hypertension-related diseases.

## 7 Therapeutic strategies to target CXC chemokines in PE and GDM

Wu et al. (2016) reported that CXCR2 regulated the expression of p-Akt, MMP-2, and MMP-9. CXCR2 inhibition with MK-2206 (2HC) suppressed trophoblast invasion by downregulating MMP-2 and MMP-9 levels via the Akt signaling pathway. CXCR2 inhibition reduced the inflammatory mediators such as MMP-2 and MMP9 during PE. In the placenta, nobiletin-added combination therapy also suppressed placental AKT and activated MAPK ERK1/2 during GDM. It also inhibited expression of CXCL8 secretion in placental tissue and VAT (Lacorte et al., 2022). These findings suggest that nobiletin works by activating the NF-kB, AKT, and MAPK/ERK1/2 pathways to enhance insulin sensitivity in tissues that have been impaired by inflammation and proinflammatory chemokines.

Furthermore, since CX3CL1 levels in the blood are linked to indicators of insulin resistance in people with GDM, researchers investigated whether resveratrol therapy might reduce CX3CL1/CX3CR1 production. Interestingly, in light of their results, the authors reported the therapeutic effects of resveratrol on reducing hyperglycemia and reductions in CX3CL1 production (Szukiewicz et al., 2017). Thus, pregnant women with metabolic disorders and euglycemia should benefit most from resveratrol’s anti-inflammatory effects on placental CX3CL1/CX3CR1 signaling. A comprehensive summary of the subsequent clinical and laboratory CXC chemokine and receptors (CXCRs) and CX3CL1 investigations in PE and GDM can be found in Table 4. In a few clinical and experimental studies on PE and GDM, CXC chemokines and receptors CXCRs and CX3CL1 are being investigated. The techniques required to target CXC chemokines and receptors CXCRs and CX3CL1 will reveal their potential use in PE and GDM.

**TABLE 4:** CXC and CX3CL1 chemokines inhibitors and target strategies in PE and GDM.

Inhibitor/Drug	Target	Disease	Outcome/Result	References
Inhibitor BRD4 (JQ1)	CXCL1/GRO-α	PE	BRD4 inhibitor decreases the expression of chemokine (CXCL1) in primary trophoblasts and in HUVECs	Liong et al. (2018)
Allopurinol	CXCL1and CXCL8	PE and GDM	Allopurinol reduced the additional pro-inflammatory chemokines (CXCL1 and CXCL8) responses to excess glucose	Negi et al. (2020)
miR-210-3p	-	-	Overexpressing of miR-210-3p in HTR8/SVneo decreased the mRNA levels of invasion and enEVT differentiation marker especially CXCL1	Hayder et al. (2021)
STOX1 Mutation	CXCL6/GCP-2	-	STOX1 Mutation in EVT negatively impacts decidual angiogenesis and immune cell	Dunk et al. (2021)
			recruitment (CXCL6)	
ISG15	CXCL8/IL-8	-	ISG15 silencing enhanced levels of IL-1β-induced pro-inflammatory cytokine CXCL8 in HTR8/SVneo cells	Ozmen et al. (2022)
Inhibitor SB203580	-	-	MAPK kinase, p38 kinase, and NF-kB inhibitor suppressed production of CXCL8	Li et al. (2011)
BAY11-7082,5				
Inhibitor BRD4 (JQ1)	-	-	BRD4 inhibitor decreases the expression of CXCL8 in primary trophoblasts and HUVECs	Liong et al. (2018)
Inhibitor	-	-	Blockade of TGF-b increased co-expression of CXCL8	Zhang et al. (2019)
SB431542				
Inhibitor	-	-	HCG induced CXCL8 mRNA expression was decreased by a JNK inhibitor	Kiyokoba et al. (2022)
SP600125				
Inhibitors	CXCL10/IP-10	-	p38 and JNK inhibitors significantly inhibited hCG-induced CXCL10 mRNA expression in THP-1 cells	Kiyokoba et al. (2022)
SB202190				
Inhibitors	-	-	In HTR-8/SVneo, p38 MAPK, PI3K, and JAK inhibitors greatly decreased the production of CXCL10 that was promoted by IL-27	Yin et al. (2014)
SB203580 LY294002				
AG490				
EV-encapsulated miR-101	CXCL11/I-TAC	-	EV-encapsulated miR-101 specifically inhibits the expression of the NF-kB/CXCL11 by downregulating BRD4 expressions	Cui et al. (2020)
AMD3100	CXCL12/SDF1-α/CXCR4	-	dMSCs treated with CXCR4 inhibitor AMD3100 significantly decreased	Lei et al. (2019)
miR-141	-	-	Hypoxia induces miR-141, which promotes apoptosis and inhibits HTR-8/SVneo cell invasion and vascularization via reducing CXCL12β and CXCR2/4 signaling pathways	Wu et al. (2019)
Inhibitors PD184325 SP600125	CX3CL1	-	MEK1/2, JNK, and NF-kB inhibitors suppressed CX3CL1 production in first-trimester decidual cells during PE	Huang et al. (2019)
SB203580				
BAY11-7082				
α-PI3K, wortmannin				
Naringenin	CXCL1 and CXCL8	GDM	Naringenin decreases the expression of pro-inflammatory chemokines (CXCL1 and 8) in the placenta and VAT	Nguyen-ngo et al. (2019)
Nobiletin	CXCL8	-	Nobiletin treatment decreased TNF-induced production of CXCL8	Chen et al. (2019), Nguyen-ngo et al. (2020a)
Punicalagin and curcumin	CXCL1,5 and 8	-	Punicalagin and curcumin were also found to significantly reduce the expression of the pro-inflammatory chemokines CXCL1,5, and 8	Nguyen-ngo et al. (2020b)
Selenium	CXCL1, 5, 8, and 10	-	Pre-treatment with selenium significantly reduced the expression of pro-inflammatory chemokines (CXCL1, 5, 8, and 10)	Nguyen-ngo et al. (2022)
SCFAs butyrate and propionate	CXCL1, 2, 5, 8, and 10	-	SCFAs butyrate and propionate inhibited TNF and LPS-induced inflammatory chemokines (CXCL1, 2, 5, 8, and 10) in placenta, VAT, and SAT tissue of GDM	Roy et al. (2020)
Resveratrol therapy	CX3CL1/CX3CR1	-	Resveratrol’s anti-inflammatory effects on placental CX3CL1/CX3CR1 signaling	Schiano et al. (2019)

## 8 Conclusions and future perspectives

We tried to clarify the expression, underlying molecular processes, origins, and essential roles of CXC and CX3CL1 chemokines in PE and GDM. In particular, CXCL1, 8, 10, 12, 16, and CX3CL1 play a vital role in the inflammation, endothelium dysfunctions, IR, and antiangiogenic processes during PE and GDM. In the studies mentioned above, PE and GDM patients had altered CXC and CX3CL1 chemokines. This review paper summarized the latest CXC and CX3CL1 chemokine molecular mechanisms underlying PE and GDM pathophysiology. This enabled insights into future difficulties and possibilities for distinct clinical and therapeutic properties. Consequences for PE and GDM, CXC and CX3CL1 chemokines inhibitors or drugs prevent the emergence and occurrence of severe PE and GDM, which may aid in improving the diagnosis and treatment of PE and GDM.

However, our knowledge of the complicated communication mechanism between CXC and CX3CL1 chemokines and their receptors is still lacking, which may limit the development of novel PE and GDM treatments. Each chemokine and receptor should be fully and immediately addressed to guarantee therapeutic relevance. Thus, more clinical and preclinical studies are needed to determine the molecular mechanism and whether an anti-inflammatory strategy targeting CXC and CX3CL1 chemokines and receptors could prevent PE and GDM progression.

## Figures and Tables

**Table:** 

**PE**	preeclampsia
**GDM**	gestational diabetes mellitus
**HER2**	human epidermal growth factor receptor 2
**CC**	chemokines with a C-C motif
**XC**	chemokines with an X-C motif
**CXC**	chemokines with a C-X-C motif
**CXXXC**	chemokines with a C-X3-C motif
**CXCR**	C-X-C chemokine receptor
**CXCLs**	CXC chemokines
**NK**	natural killer
**TNF-α**	tumor necrosis factor-alpha
**MAPKs**	mitogen-activated protein kinases
**JAK**	Janus kinases
**STAT**	signal transducer and activator of transcription
**p-Akt**	phosphorylated- Ak strain transforming
**GRO-α**	growth-related oncogene protein
**IL-8**	interleukin *-*8
**NF-kB**	nuclear factor kappa B
**VEGF**	vascular endothelial growth factor
**IL1-β**	interleukin-1 beta
**ERK1/2**	extracellular signal-regulated kinase1/2
**EVT**	extravillous trophoblasts
**MMP2 and 9**	matrix metalloproteinase 2 and 9
**BET**	bromodomain and extra-terminal
**BRDs**	bromodomains
**HUVECs**	human umbilical vein endothelial cells
**IL-1**	interleukin-1
**HELLP**	hepatic transaminases low platelets
**DEGs**	differentially expressed genes
**TARID**	TCF21 antisense RNA-inducing promoter demethylation
**STOX1**	STORKHEAD-BOX1 PROTEIN 1
**MiR**	microRNA
**PI3K/AKT**	phosphatidylinositol 3-kinase/protein kinase B
**HDAC2**	histone deacetylase 2
**FOXA1**	forkhead box protein A1
**ISG15**	interferon-stimulated gene 15
**GPCR**	G-protein-coupled receptor
**dNK cells**	decidua natural killer cells
**DCs**	dendritic cells
**TGF-β**	transforming growth factor-β
**LncRNAs**	long non-coding RNAs
**EVs**	extracellular vesicles
**circRNAs**	circular RNAs
**dMSCs**	decidual mesenchymal stem cells
**STBs**	syncytiotrophoblasts
**DSCs**	decidual stromal cells
**PBMC**	peripheral blood mononuclear cells
**FGR JNK**	fetal growth factor jun N-terminal kinase
**GCP-2**	granulocyte chemotactic protein 2
**hCG**	Human chorionic gonadotrophin
**IFN-γ**	interferon-gamma
**IP-10**	human interferon-inducible protein 10
**I-TAC**	interferon-inducible T-cell alpha chemoattractant
**SDF1-α**	stromal cell-derived factor 1- alpha
**LPS**	lipopolysaccharide
**VAT**	visceral adipose tissue
**SAT**	subcutaneous adipose tissue
**SCFAs**	short-chain fatty acids
**GDMM**	gestational diabetes mellitus mother
**NPM**	normal pregnancy mother
**T1D**	type 1 diabetes
**T2D**	type 2 diabetes
**CMSCs**	human chorionic membrane-derived stem cells
**STBs**	syncytiotrophoblast
**FMT**	fecal microbiota transplantation
**KISS1 and KISS1R**	kisspeptin-1 and kisspeptin-1 receptor
**IGF**	insulin growth factor
**UACR**	urine albumin-to-creatinine ratio
**BMI**	body mass index
